# A Microfluidic Chip and a Portable Colorimetric Detection Device for the Rapid, Low‐Cost, and Accurate Diagnosis of ASFV

**DOI:** 10.1155/tbed/9794229

**Published:** 2026-06-18

**Authors:** Wenju Ren, Rui Yang, Chenyang Qi, Jie Zhou, Liu Yang, Yuandi Yu, Taixiong Zheng, Lizhi Fu

**Affiliations:** ^1^ School of Integrated Circuits, Chongqing University of Posts and Telecommunications, Chongqing, 400065, China, cqupt.edu.cn; ^2^ Chongqing Academy of Animal Science, Chongqing, 402460, China; ^3^ National Center of Technology Innovation for Pigs, Chongqing, 402460, China; ^4^ Rongchang, Animal Disease, Observation and Research Station, Ministry of Agriculture and Rural Affairs, Chongqing, 402460, China, agri.gov.cn

**Keywords:** ASFV, LAMP, microfluidic chip, on-site inspection, portable diagnostics

## Abstract

The lack of simple, rapid, accurate, and low‐cost on‐site diagnostic technology has seriously hindered the effective prevention and control of major infectious diseases such as African swine fever (ASF). This study presents an integrated, portable diagnostic system comprising a low‐cost microfluidic chip and a fully automatic, portable device for simultaneously detecting nine samples. The aim is to achieve rapid on‐site detection of African swine fever virus (ASFV) nucleic acid. The system integrates magnetic bead nucleic acid extraction, transfer, loop‐mediated isothermal amplification (LAMP), and optical detection functions, and can perform fully automatic, closed‐tube analysis of 9 samples in 25 min, effectively avoiding aerosol pollution. The detection limit for the ASFV *P10* gene is 10^2^ copies/μL. In a blind test involving 87 clinical samples, the system demonstrated excellent performance with a clinical sensitivity and specificity of 97.6% (42/44) and 98.0% (42/43), respectively, and an accuracy of 96.6% (84/87). When the sample concentration exceeds the detection limit, sensitivity can reach 100 %. The core advantages of this method are that it is fast, accurate, and low cost, and does not require professional laboratories or operators. Its modular design makes it easy to expand to a 100‐channel, high‐throughput detection system at low cost. This system provides a powerful technical tool for large‐scale epidemic screening and prevention in areas with limited resources, offering significant public health and economic value.

## 1. Introduction

In recent years, the frequent emergence infectious diseases (EIDs) amid globalization, urbanization, and climate change have posed persistent and severe challenges to public health security, economic stability, and food safety [1, 2]. Among these, zoonotic diseases constitute the overwhelming majority of EIDs, underscoring the critical importance of the “One Health” concept, which recognizes the inextricable link between animal and human health [3, 4]. Certain transmissible zoonotic pathogens capable of spreading across multiple species may pose far greater threats to human society than viruses confined to a single host. These pathogens are increasingly becoming the greatest public health threats, as evidenced by events such as avian influenza, the COVID‐19 pandemic, and Ebola virus disease (EVD) not only reveal the potential risks of pathogen transmission across species but also expose global deficiencies in early infectious disease surveillance and rapid response capabilities [2, 5, 6]. Their spread can trigger social panic, undermine societal stability, and pose challenges to public health security. Against this backdrop, developing rapid, accurate pathogen diagnostic technologies suitable for resource‐constrained settings, such as grassroots veterinary stations, and border checkpoints in developing countries, has become a critical scientific frontier and urgent necessity. Such technologies are essential for effectively controlling outbreak spread, assessing infection risks, guiding epidemic prevention and control to minimize economic losses, and safeguarding food supply chain security [7].

African swine fever (ASF) is a highly contagious and lethal viral hemorrhagic disease of pigs caused by the African swine fever virus (ASFV). With a mortality rate of up to 100%, ASF has had a devastating impact on the global pig husbandry [8, 9]. Although ASFV has not yet demonstrated effective human‐to‐human transmission, the immense economic losses and meat supply crises it causes severely threaten the livelihoods of millions of farmers worldwide and threaten regional food security [10]. In the absence of commercially available vaccines, controlling the disease relies heavily on biosecurity measures such as early diagnosis, strict quarantine, and rapid culling [11]. Therefore, establishing rapid and accurate diagnostic methods that can be implemented immediately at outbreak sources, such as farms and markets, is crucial for breaking the ASFV transmission chain and achieving effective control [12, 13].

Despite significant advances in molecular diagnostic technologies over the past few decades, it remains challenging to translate these methods from well‐equipped central laboratories to field settings. The current gold standard laboratory diagnostics for ASFV include virus isolation, the enzyme‐linked immunosorbent assay (ELISA), and real‐time quantitative polymerase chain reaction (qPCR) [14–18]. qPCR is the most widely adopted method due to its exceptional sensitivity and specificity. However, these methods rely heavily on sophisticated instruments, specialized operators, and lengthy testing cycles, as well as stable laboratory environments [19]. This renders them unsuitable for rapid field screening, which requires timeliness, portability, and low cost.

Loop‐mediated isothermal amplification (LAMP) technology is an emerging nucleic acid amplification technique that is regarded as an ideal platform for point‐of‐care molecular diagnostics. This is due to its simplicity, rapid reaction time (typically 15–60 min), and relatively lenient requirements for nucleic acid extraction purity [20, 21]. LAMP reactions proceed at a constant temperature of ~60–65°C and require only a simple heating block, thus eliminating the need for expensive thermal cyclers. However, traditional LAMP detection often relies on endpoint turbidity observation or dye color changes, which can be subjective and difficult to quantify accurately. These limitations constrain its potential in applications requiring precise quantification or high throughput.

Microfluidic technology offers a revolutionary, fully automated diagnostic approach where samples are input and results are output by manipulating fluids at the microliter to nanoliter scale to integrate complex biochemical operations at the chip scale [22–24]. Researchers have successfully integrated multiple steps, such as nucleic acid extraction, purification, amplification, and detection, onto microfluidic chips, thereby significantly reducing the risk of human error and cross‐contamination [25]. However, most highly integrated microfluidic systems reported in the literature rely on complex and costly fabrication processes, such as soft photolithography, and require bulky external pump and valve control systems [26]. This results in high manufacturing costs and poor portability, hindering large‐scale deployment and point‐of‐care implementation.

In point‐of‐care testing, the ability to reliably interpret results is paramount. The widespread adoption of smartphones provides robust hardware support for the development of low‐cost, portable testing platforms [27, 28]. Colorimetric testing using smartphone cameras has received a lot of attention due to its intuitive nature and low inexpensive. However, variations in ambient light, differences in camera performance, disparities in photo processing software, and the absence of effective built‐in calibration frequently result in unstable color measurements and poor reproducibility, severely compromising diagnostic accuracy and reliability [29]. Meanwhile, fluorescence measurements require complex optical designs, including dichroic beam splitters, specific filters, and excitation light sources with precise wavelengths [30, 31]. This poses significant challenges to miniaturizing devices. Furthermore, the need for complex I–V circuits to amplify fluorescence signals limits the application of fluorescence measurements [32–34]. These factors contribute to the high cost of fluorescence detection methods. Given the need for low cost and ease of use, colorimetry is used for result diagnosis.

The core bottleneck in the development of current ASFV field diagnostic technologies lies in how to integrate highly efficient sample pretreatment (nucleic acid extraction and transfer), highly sensitive LAMP amplification, and stable, reliable colorimetric detection readout into a single, truly low‐cost, portable, and fully automated device. In order to address the aforementioned challenges, this study has designed and developed an integrated, rapid nucleic acid diagnostic device. The device’s core innovation lies in its fusion of multiple technologies, which aims to enable the rapid, low‐cost, and accurate on‐site diagnosis of the ASFV.

A compact, fully automated, microfluidic chip that is functionally integrated has been designed. The system employs a simplified superhydrophobic design and uses silicone oil for magnetic bead transfer technology. It automatically and sequentially completes the entire process, from blood sample lysis and nucleic acid magnetic bead extraction, to transfer and LAMP reaction, without requiring any external pumps, valves, or manual intervention.

Instead of using traditional, costly microfabrication techniques, a low‐cost manufacturing strategy is employed to produce microfluidic chips and integrated, rapid nucleic acid diagnostic devices. The microcomputer numerical control (CNC) machine tools and 3D printing technologies are utilized for microfluidic chip and device hardware fabrication, with postprocessing injection molding enabling mass production. This approach significantly reduces production costs and cycle times, laying the foundation for large‐scale manufacturing.

The core of the mobile phone camera is replaced by a high‐performance, low‐cost RGB color sensor. When combined with a meticulously designed white light‐emitting diode (LED) light source driver circuit, microfluidic chip materials, and light diffusion films, this configuration establishes a stable, ambient light‐interference‐free integrated colorimetric detection module that enables consistent colorimetric quantification. Rigorous white balance calibration and signal processing algorithms ensure precise quantitative analysis of LAMP reaction products.

The integration of microfluidic chips, temperature control systems, optical detection module, and control systems into a single, portable, 2 kg device achieves a truly “all‐in‐one” and portable design that fulfills the ultimate goal of “sample in, result out”. This offers significant advantages for nonprofessional users operating in environments with limited resources.

This paper will detail the design, fabrication, and performance evaluation of this integrated portable point‐of‐care testing device (POCT). The materials and methods section introduces the design principles of the microfluidic chip, the hardware configuration of the device, the manufacturing process, and the biochemical reagents employed. The results and discussion section will systematically present and discuss the validation of the device’s automated workflow feasibility, the stability and reproducibility of the colorimetric detection system, the assessment of nucleic acid extraction efficiency, the detection limit analysis for ASFV, and the diagnostic performance based on clinical samples, including sensitivity, specificity, and accuracy. Finally, this paper will summarize the core contributions of the study and outline its application prospects and future development directions.

This study not only provides a powerful technical tool for ASFV prevention and control but also offers a replicable technical paradigm and design approach for developing integrated, low‐cost point‐of‐care molecular diagnostic platforms for other major infectious diseases.

## 2. Materials and Methods

### 2.1. Plasmids and Clinical Samples

In this study, the ASFV *p10* gene is selected as the detection target. The *p10* gene encodes the viral capsid protein and is highly conserved among various ASFV epidemic strains, with a mutation rate significantly lower than that of genes such as *p72* and *p54*, thereby ensuring broad applicability of the detection method [35]. Sequence homology analysis indicates that the *p10* gene has no significant homologous sequences with other common pathogenic microorganisms of porcine origin, demonstrating good detection specificity [36]. Furthermore, previous studies have confirmed that isothermal amplification‐based detection methods using the *p10* gene are stable and reliable, providing a solid foundation for the selection of targets and the development of methods in this study.

This study employed two types of samples for experimental evaluation. The first type is the standard plasmid sample; a 300‐bp target gene fragment is synthesized based on the nucleotide sequence of the ASFV *p10* gene in GenBank (accession number: X68563.1) and cloned into the pUC57 vector [34]. Standard plasmid samples are subjected to serial dilutions using ultrapure water, and their concentrations are determined using a NanoDrop one microvolume spectrophotometer (Thermo Fisher Scientific, USA). Subsequently, based on Avogadro’s constant, the mass concentration is converted to copy number concentration using Equation (1):

(1) copy numbercopies/µL=A2606.021023ng/µL×10−9××DNA lengthbp×660

The second category consists of blood samples collected from specific pathogen‐free (SPF) pigs and pigs infected with ASFV (confirmed by qPCR) that are stored in the laboratory.

Porcine whole blood is selected as the detection sample in this study based on the following considerations: First, blood is one of the most readily accessible sample types from live pigs, with collection via ear vein being minimally invasive and operationally convenient, meeting the requirements of point‑of‑care testing. Second, viral loads in blood during the acute phase of ASFV infection are high, facilitating early diagnosis [37]. Third, whole blood samples can be directly processed in the lysis chamber of the microfluidic chip without complex pretreatment such as tissue homogenization, making them highly compatible with the integrated detection device [38]. Finally, we have verified that the blood matrix does not significantly affect detection performance, with the limit of detection remaining at 10 copies/μL. These samples are collected, diagnosed, and stored by the Chongqing Academy of Animal Sciences (CQAAS). For the experiment, the ASFV‐positive blood samples are diluted 1000‐fold. Healthy samples are uniformly mixed with synthetic plasmids. Samples are formed by combining the samples with nucleic acid extraction reagents at a ratio of 1:1 (15: 15 μL). The CQAAS has synthesized plasmid samples of other viruses that share common hosts with ASFV, such as the classical swine fever virus (CSFV), the porcine reproductive and respiratory syndrome virus (PRRSV), the porcine epidemic diarrhea virus (PEDV), and the pseudorabies virus (PRV), for use in specific detection [34].

First, dilute the synthetic plasmid to 1 × 10^6^ copies/μL. Then, take 10 μL of the diluted plasmid sample and mix it with 90 μL of heparinized SPF pig whole blood (donated by the CAAS Veterinary Research Institute) to obtain a plasmid solution at a concentration of 1 × 10^5^ copies/μL. Subsequently, perform a 10‐fold serial dilution of the sample using SPF pig whole blood to prepare a gradient of plasmid concentrations ranging from 1 × 10^0^ to 1 × 10^5^ copies/μL for subsequent detection.

### 2.2. LAMP Reaction System and Materials

To perform LAMP detection, specific primers are designed and synthesized according to the protocol reported, as shown in Table 1 [39]. Subsequently, an amplification system is constructed using the LAMP reaction premix from Sangon Biotech (Shanghai) Co., Ltd., and the specificity and sensitivity of this system are validated. Genomic DNA from all samples in the experiment is extracted using the Universal Genomic DNA Extraction Kit from Tiangen Biotech (Beijing) Co., Ltd.

**Table 1:** Primer sequence table for ASFV‐LAMP method.

Item	Nucleotide sequence
F3	AAATGGCACTCCACTTCC
B3	AATATGGCTTGAATTTCTGGT
BIP	CAGGCAAAACAAGTGAAACACCTTTTTAGTATTCGAGGATGCCCAT
FIP	GACTCGTTTGTTCATTATTCGTGTTTTTTAAAAGACAACAATTAAGGAGGC
LB	AAAAAATCCCACGAATGCGATGTTC

The lamp reaction system is shown in Table 2 [34]. Calcein solution (Aladdin) is used as the chromogenic agent in the experiment. The total volume of the entire reaction system is 50 μL.

**Table 2:** Composition of LAMP system.

Item	Volume (μL)
MIX	25
FIB	4
BIP	4
LB	2
F3	1
B3	1
Bst DNA Enzyme	1
H_2_O	7
Calcein	5

Analyze nucleic acid amplification results using electrophoresis technology and visualize electrophoresis results using a UVP ChemStudio multifunctional imaging device (Analytik Jena GmbH). Quantitative analysis of qPCR nucleic acid molecules is performed using the QuantStudio 5 (Thermo Fisher Scientific).

### 2.3. Diagnostic Principle Based on Color Changes Mediated by Nucleic Acid Amplification Byproducts

Two colorimetric reagents are commonly used in colorimetric measurements: hydroxynaphthol blue (HNB) and calcein [35–37]. Among them, HNB changes from purple to sky blue during the amplification reaction, exhibiting relatively low contrast. This color shift is difficult to distinguish and relies heavily on subjective judgment, making it unsuitable for automated quantification. In contrast, calcein produces intense yellow‐green fluorescence after amplification, delivering high signal intensity. Even weakly positive samples are easily distinguishable, rendering it particularly suitable for semiquantitative and qualitative analysis [40]. To ensure reliable and stable results, calcein is selected for the study due to its stronger signal and easier analysis.

In the standard LAMP reaction system, manganese ions (Mn^2+^) and calcein are added. As a metal fluorescent indicator with fluorescence, calcein preferentially binds with Mn^2+^ in its initial state, forming a stable complex (calcein‐Mn^2+^). This complex quenches the fluorescence of calcein itself, meaning that the system is usually an extremely light yellow before the reaction.

**Figure 1:**
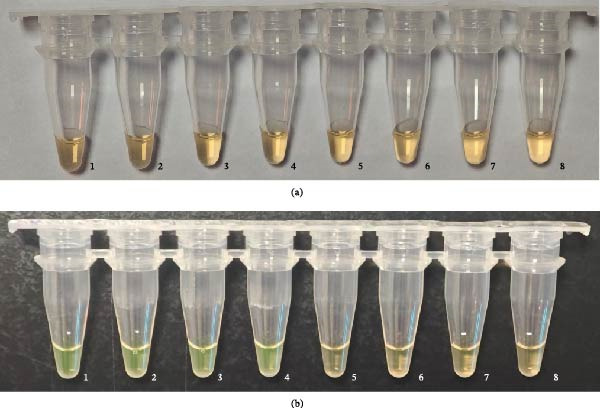
Visualization of LAMP amplification results based on calcein: (a) the reaction tube before amplification, (b) the reaction tube after amplification. 1: 10^6^ copies/μL; 2: 10^5^ copies/μL; 3: 10^4^ copies/μL; 4: 10^3^ copies/μL; 5: 10^2^ copies/μL; 6: 10^1^ copies/μL; 7: 10^0^ copies/μL; 8: ultrapure water.

During LAMP amplification, DNA polymerase releases a pyrophosphate radical (PPi) for each dNTP added. The large quantity of PPi produced by extensive amplification combines with Mg^2+^ in the reaction system to form insoluble magnesium pyrophosphate (Mg_2_P_2_O_7_) precipitates. Importantly, Mn^2+^ has a much higher affinity for PPi than for calcein. Consequently, once Mg_2_P_2_O_7_ is formed, calcein is released from the calcein‐Mn^2+^ complex. The released calcein then combines with free magnesium ions (Mg^2+^) to form a complex with strong yellow‐green fluorescence (calcein‐Mg^2+^). The results of the reaction are shown in Figure 1.

### 2.4. Design and Fabrication of Microfluidic Chips

In order to reduce the complex manual processes involved in nucleic acid lysis, transfer, and amplification, and to enable the technology to be used in both on‐site and remote areas, a low‐cost microfluidic chip has been developed to automate sample processing and detection. Due to its high mechanical strength, excellent weather resistance, low cost, and compatibility with various processing methods, such as CNC machining, laser engraving, thermal stamping, and injection molding, polymethyl methacrylate (PMMA) is used in applications ranging from prototyping to mass production. A desktop‐level CNC machining center is used to produce microfluidic chips from PMMA. In the later stage, injection molding can be used to achieve low‐cost, large‐scale production.

However, due to PMMA’s high sensitivity to organic solvents such as ethanol and acetone, these are excluded from the fabrication of the microfluidic chips and the reagents. At the same time, the chips undergo simple hydrophobic treatment, and silicone oil is used as a continuous phase to generate a “water‐in‐oil” structure. This protects PMMA from exposure to reagents and other substances. Consequently, it improves the reliability and weather resistance of PMMA microfluidic chips.

**Figure 2:**
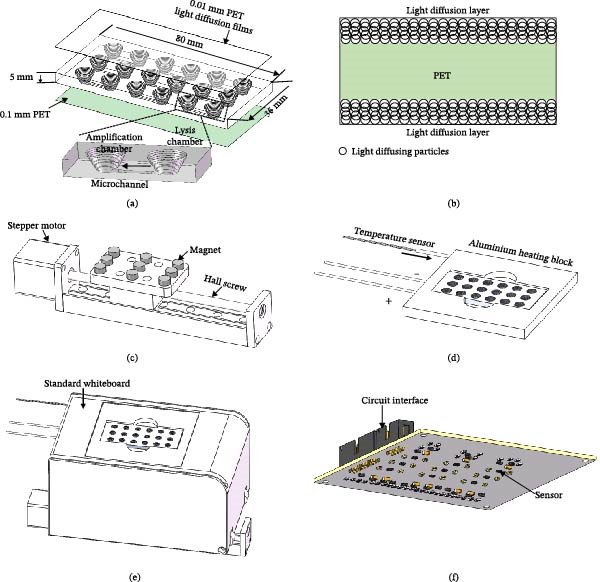
(a) The structure of microfluidic chip, (b) the PET light‐diffusing film, (c) the structure of magnetic bead transfer device, (d) the structure of heating plate, (e) the structure of the magnetic bead transfer and heating device, and (f) diagram of the sensor module.

Figure 2a shows the design of the microfluidic chip. It can be divided into three layers: the middle layer is the chamber layer, and the top and bottom layers are the sealing layers. For the convenience of color measurement, the top protective films are 0.1‐mm‐thick polyester (PET) light diffusion films, the structure of which is shown in Figure 2b. In addition to its sealing function, the PET light diffusion film can transform highly directional and nonuniform spatial light intensity distribution into a low‐direction and statistically uniform surface light source via a microscopic optical scattering mechanism, thereby minimizing radiation flux loss and improving the accuracy of color measurement.

The microfluidic chip is composed of nine sample lysis chambers and nine amplification chambers, which are integrated into nine distinct groups. Each group consists of one sample lysis chamber and one amplification chamber. The lysis chambers are employed for the purpose of sample lysis, and magnetic beads are then used to adsorb the resulting products, forming magnetic bead nucleic acid complexes. The transfer process is initiated by the utilization of a microchannel connection between two chambers by the magnetic bead nucleic acid complex, as illustrated in Figure 2a. In the amplification chamber, the magnetic bead‐nucleic acid complex is first desorbed. This is followed by the amplification of the target nucleic acid and the detection of the results of this amplification.

Following preliminary optimization, the dimensions of the chip are set at 80 mm in length and 30 mm in width. The lysis chamber and amplification chamber have been designed as circular frustum structures, with diameters of 4 and 8 mm at both ends of the frustum, respectively. The inverted circular frustum structure has been shown to facilitate the accurate positioning of reagents and enhance the accuracy of nucleic acid amplification detection in subsequent stages of the process. The width and height of the microchannel are 3 and 0.25 mm, respectively. The volume of the two chambers and channels is 293.65 μL.

PET light diffusion film and optical grade double‐sided tape (Shenzhen Hongzhan Optoelectronic Technology Co., Ltd) is used to stick to sealing the chambers. The chamber layer is bonded to the upper and lower PET films using optical‐grade double‐sided adhesive tape. This tape features a polyethylene terephthalate (PET) substrate coated on both sides with acrylic pressure‐sensitive adhesive, offering excellent bonding strength, thermal stability (long‐term resistance up to 150°C), and chemical stability. It also exhibits residue‐free and waterproof characteristics. During lamination assembly, the tape thickness (30 μm) and its ~15% elastic compression deformation result in a net impact of only 4.5 μm on the microfluidic channel height (250 μm), accounting for 1.8% of the total height. This is far below the substrate thickness tolerance (±100 μm), rendering its effect on the chip’s overall dimensional accuracy and functionality negligible. According to ASTM D3330 standards, the 90° peel strength test of pressure‐sensitive tape yielded an adhesion strength of 109 N/100 mm [39, 41]. This result indicates that the tape provides reliable bonding strength for interlayer bonding of microfluidic chips.

To achieve robust bonding between the microfluidic chip chamber layer and the cover, base layer, the following surface treatment and bonding process is employed. First, the chamber layer surface is thoroughly cleaned with isopropyl alcohol to remove contaminants such as grease and fingerprints. Subsequently, the surface undergoes a 1 min plasma cleaning and activation treatment. Following this, the activated chamber layer surface is modified using a silane coupling agent. This coupling agent forms strong covalent bonds between the polar groups on the PMMA surface and the acrylic pressure‐sensitive adhesive. Simultaneously, it constructs a hydrophobic siloxane network at the interface, effectively blocking water molecule penetration and resisting acid, alkali, and solvent erosion. This significantly enhances the interface’s adhesion, water resistance, and long‐term durability [27, 28].

Chip bonding is performed according to the manufacturer’s technical specifications. First, 100 kPa of pressure is applied using a roller, which is rolled back and forth three times to ensure full contact between the layers and eliminate initial bubbles. The preliminarily bonded chip is then placed in a vacuum chamber for degassing to remove any residual gases and complete the sealed bond. Finally, the chip is left to cure at room temperature for 72 h, which allows the adhesive to reach its maximum bonding strength.

To ensure the sealing integrity and pressure tolerance of the microfluidic chip, a pressure sensor and syringe pump are used to evaluate it after fabrication. Ultrapure water is used as the working fluid during the evaluation process [16]. The experimental results showed that the fabricated microfluidic chip could withstand pressures of up to 41 kPa without leaking. This suggests that the bonding strategy designed for this purpose is effective in preventing leakage under operational conditions.

It is imperative that the two chambers and the channel undergo a hydrophobic treatment. In the experiment, a brush and a superhydrophobic agent are utilized to perform a simple hydrophobic treatment on the two chambers and microchannels of the microfluidic chip. Following the hydrophobic treatment, the material should be allowed to dry for a period of 1 h in order to facilitate the formation of a stable hydrophobic film. Following the hydrophobic treatment, the isolation of the chambers is possible, whilst permitting the transfer of magnetic beads between them without leaving any residue.

Due to its good biocompatibility, silicone oil does not cause toxicity or adverse effects on biological samples such as cells and proteins. Concurrently, silicone oil exhibits good optical transparency, thereby enabling unobstructed light propagation within microfluidic channels and facilitating color measurement. Therefore, silicone oil is selected as the continuous phase in microfluidic chips to generate water droplets in oil for detection. At the same time, it serves to isolate the individual chambers, thereby preventing direct contact and cross‐reaction between them. After chip fabrication, red and blue dyes are added to the chips. They are left undisturbed for 1 week to test the chips’ hydrophobicity, isolation performance, and sealing integrity. The chip exhibits no leakage and possesses excellent isolation performance, as illustrated in Figure S1.

After completing the fabrication of the microfluidic chip, add 100 μL of silicone oil to both the lysis chamber and the amplification chamber. Then add 37.65 μL of quick DNA extraction solution and 3 μL of magnetic beads to the lysis chamber. The extraction solution and magnetic beads will converge at the bottom of the chamber for sample pretreatment and lysis.

Preload 50 μL of LAMP reaction system into the amplification chamber. After the addition is complete, the reaction system will be established and finally sealed with PET light diffusion film. The chip can be stored at –18°C. Under these conditions, the chip can be stored for 12 months.

### 2.5. Design of Magnetic Bead Transfer Mechanism

As shown in Figure 2c,e, a permanent magnet‐driven microfluidic system has been designed to meet the requirements of portability, reduced system complexity, and low cost. The system can efficiently transfer magnetic bead nucleic acid complexes without the need for external pumps, valves, or other external equipment, which greatly reduces the complexity and cost of the system while improving its reliability. The system uses cylindrical NdFeB magnets with a diameter of 5 mm, a height of 10 mm, and a residual magnetism of 1.2 T for magnetic bead transfer. The working temperature is ≤80°C, and the Curie temperature is 350°C, enabling reliable operation at the temperature of LAMP amplification. The NdFeB cylindrical magnet generates sufficient magnetic force at a distance of 3 mm from the chip surface to overcome the viscous resistance of Fe_3_O_4_ magnetic beads in lysis solution, silicone oil, and amplification solution.

A permanent magnet support has been designed based on the microfluidic chip to fix nine cylindrical permanent magnets, due to the need to transfer nine samples simultaneously in microfluidic chips. Once the magnets have been fixed in place, attach the support to the nut seat of the ball screw as shown in Figure 2c. The nut seat is controlled by a stepper motor to move at a constant speed of 0.5 mm/s, driving the magnetic beads along a 250 µm high channel. The entire transfer process takes 15 s. Once the magnetic bead transfer is complete, there is no visible residue in the microchannel.

To validate magnetic bead transfer efficiency, a microfluidic chip chamber layer is fabricated using transparent PMMA. A camera is used to observe the entire microchannel before and after bead transfer.

Verification of magnetic bead transfer efficiency via microphotography revealed no significant residual beads in the lysis chamber when transferred at a flow rate of 0.5 mm/s from the lysis chamber to the amplification chamber, as shown in Figure S2a and b. This demonstrates that the designed microfluidic chip and POCT system can meet the requirements for rapid point‐of‐care testing.

### 2.6. Heating Device and Temperature Control System

The system’s heating device comprises an aluminum heating block and a 100 W flexible polyimide heating film, which is powered by a 24 V supply. The heating film is 0.3 mm thick, and the groove for installing the microfluidic chip is designed to fit the chip’s dimensions. The microfluidic chip and aluminum heating block feature a clearance fit to facilitate the installation and removal of the microfluidic chip, with maximum and minimum clearances of 0.10 and 0.05 mm. The heating plate has a flatness tolerance of ±0.05 mm for a 100 × 100 mm area. The aluminum heating block is manufactured using desktop CNC machining. The flexible polyimide heating film is fixed to the aluminum block using 3M double‐sided tape. To accurately measure the temperature of the aluminum heating block, drill a hole with a diameter of 2.00 mm and a depth of 10.00 mm on the side of the aluminum block for installing the temperature sensor, as shown in Figure 2d.

The system uses a self‐tuning PID control algorithm to achieve precise temperature control. Specifically, the controller is an STM32F103C8T6, a 10 kΩ negative temperature coefficient (NTC) sensor is used for temperature measurement, and a 12‐bit analog‐to‐digital converter (ADC) is used to collect temperature sensor data. A 1 kHz pulse width modulation (PWM) control MOSFET precisely adjusts the heating power of the heating film, achieving a temperature rise rate of 6°C/s and a temperature control accuracy of ±0.1°C. The controller first calculates the output control quantity u(t) based on the error *e*(*t*) between the set temperature value (SP) and the actual temperature value (PV), as shown in Equation (2).

(2) ut=Kpet+Ki∫0tetdt+Kddetdt

Then, the system’s critical gain *K*_*u*_ and oscillation period *T*_*u*_ are automatically identified using the relay feedback method, after which the PID parameters (proportional *K*_*p*_, integral *K*_*i*_, and derivative *K*_*d*_) are adjusted accordingly, as shown in Equation (3).

(3) Kp=0.6KuKi=2Kp/TuKd=KpTu/8

**Figure 3:**
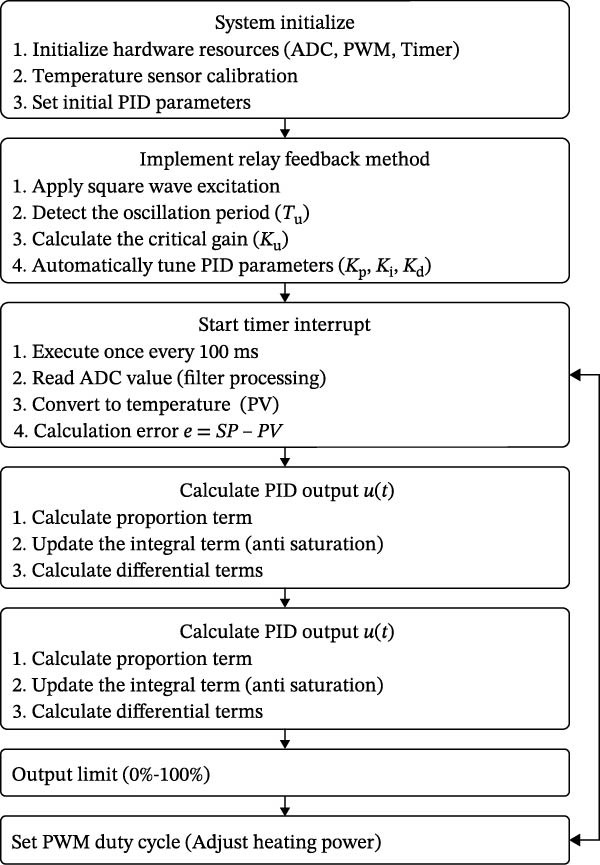
Diagram of self‐tuning temperature control algorithm.

To ensure that the controller can respond quickly and operate stably. PID calculation is performed every 10 ms during the control process. This improves the accuracy of the temperature reading and eliminates noise interference through filtering and calibration. Measures such as antiintegral saturation and temperature change rate limitation are added to the control system to enhance stability and ultimately achieve efficient and accurate temperature control. The control block diagram is shown in Figure 3.

### 2.7. Design of Optical Measurement Module

Although it is possible to use the camera on a smartphone or other smart device for color recognition and result analysis, the camera is designed for general use and is optimized for photography. The imaging effect also depends on ambient light, which leads to poor repeatability. To eliminate interference from ambient light fluctuations, external reference materials must be introduced. At the same time, complex image segmentation, color correction, and pattern recognition algorithms need to be developed. However, the above algorithm is not very universal among different smartphones and other smart devices, which makes it difficult to integrate into a stable, specialized medical device.

**Figure 4:**
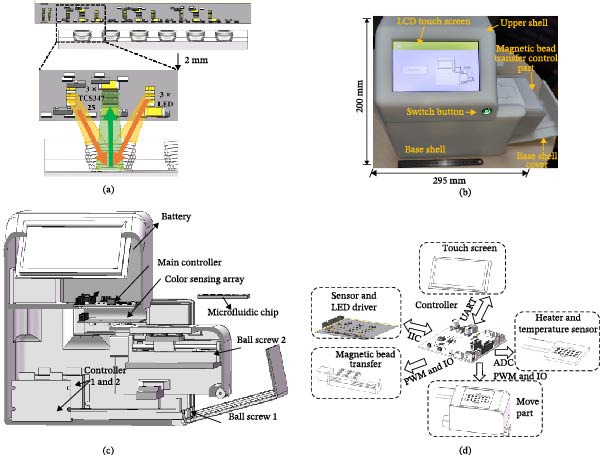
(a) Diagram of colorimetric detection, (b) the exterior appearance of POCT, (c) the internal structure of POCT, and (d) the internal connections of the device.

Equipped with built‐in red, green, and blue primary color filters and photodiodes, the color sensor can directly measure the average optical characteristics of the target area and output standardized parameters based on color space. Unlike mobile phone cameras, which rely on ambient light and require complex white balance algorithms for calibration, color sensors can provide reproducible quantitative data that is linearly correlated with visual perception. This improves system stability and reliability. At the same time, they can be integrated into the optical dark cabin of the detection equipment to form a closed, interference‐free measurement environment. This effectively prevents aerosol interference and solves issues such as the poor repeatability of mobile phone camera measurements and environmental light interference. Figure 4a illustrates the principle of color measurement using the TCS34725 color sensor.

**Figure 5:**
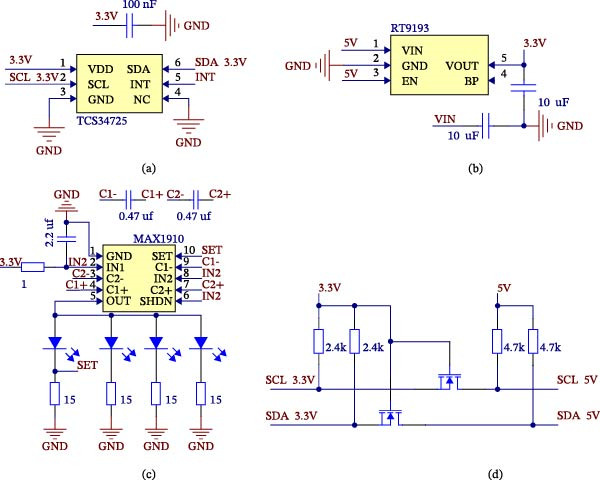
Circuit design of (a) TCS34725, (b) power conversion, (c) D65 light source, and (d) communication level conversion.

The TCS34725 color sensor is used as the detection module for color measurement in the device, providing a digital return of red (R), green (G), blue (B), and light intensity values (C). An infrared blocking filter is integrated on‐chip and localized to the color‐sensing photodiodes to minimize infrared spectral interference. The TCS34725’s high sensitivity, wide dynamic range, and infrared blocking filter make it an ideal color sensor solution [34]. The TCS34725 circuit design in the device is shown in Figure 5a.

The STM32F103C8T6 controller obtains the color measurement values via the I^2^C bus. The TCS34725 color sensor is powered by a 3.3 V power supply; therefore, an RT9193 is used in the circuit to convert the 5 V supply voltage to 3.3 V. Figure 5b shows the level conversion circuit.

The D65 light source is a standard daylight simulation with a color rendering index (CRI) of ≥95. As an artificial representation of sunlight, it is used as a benchmark for color measurement, color difference comparison, and quality control [42]. Consequently, the D65 light is used to improve color measurement. To ensure stable LED color and brightness, a precise constant current drive circuit must be designed. The device uses a dedicated LED driver chip (MAX1910) to ensure a constant power supply to the LED and maintain its stable color and brightness, as shown in Figure 3c.

Since the TCS34725 operates at 3.3 V while the STM32F103C8T6 controller operates at 5 V, a level‐shifting circuit using the BSS138 is designed to enable reliable communication between them, as shown in Figure 5d. Integrating the aforementioned circuits resulted in the sensor circuit module shown in Figure 2f. It comprises nine color detection modules, 18 LED light sources, and corresponding circuit interfaces. Figure 4d shows the relationship between the assembled color measurement circuit board and the microfluidic chip, along with the colorimetric measurement schematic diagram.

In order to meet the colorimetric analysis requirements for high‐precision nucleic acid detection, dark current compensation and white balance correction must be performed in order to eliminate sensor dark noise and color drift of the light source. To maximize the dynamic range of the measurement, the sensor gain is set to 14 and the integration time to 700 ms during the system initialization stage. At the start of the measurement process, 10 measurements should first be collected under completely shaded conditions in order to calculate the average dark current value and determine the sensor’s background noise (*R*_dack_, *G*_dack_, *B*_dack_, and *C*_dack_). Then, transfer the sensor above the standard whiteboard and take 10 measurements under illumination with a CRI > 90 to obtain the average blank values (*R*_white_, *G*_white_, *B*_white_, and *C*_white_). After deducting the dark current using Equation (4), perform white balance correction using Equation (5) to obtain the white balance coefficients (*R*_gain_, *G*_gain_, and *B*_gain_). Once calibration is complete, color changes during LAMP amplification can be measured and diagnosed using dark current values and correction coefficients.

(4) R=Rwhite−RdackG=Gwhite−GdackB=Bwhite−BdackC=Cwhite−Cdack

(5) Rgain=C/RGgain=C/GBgain=C/B

During amplification detection, 10 measurements are taken for each reaction chamber to obtain the raw RGB values (*R*_raw_, *G*_raw_, and *B*_raw_). These are then used to calculate the calibrated RGB values (*R*_final_, *G*_final_, and *B*_final_l) via Equation (6), with the calibrated final values constrained between 0 and 255. During the measurement process, preamplification and postamplification calibrated RGB values are used for result analysis.

(6) Rfinal=Rraw−Rdack/RgainGfinal=Graw−Gdack/GgainBfinal=Braw−Bdack/Bgain.

### 2.8. Design and Assembly of the POCT Device

As shown in Figure 4b, the system casing consists of a bottom shell, a partition, a bottom shell cover, and an upper shell. The bottom shell houses two ball screws, two controllers, and magnetic bead transfer components, as shown in Figure 4c. Ball screw 1 is used for the bottom shell cover switch and carries ball screw 2, which is used for transferring magnetic beads and nucleic acids for detection. The magnetic bead transfer component comprises a heating block and a ball screw housing, see Figure 4d. The shell is 3D‐printed (Bambu P1S) using environmentally friendly polylactic acid (PLA).

The main controller, color sensing array, and 24 V lithium‐ion battery are installed on the partition. As shown in Figure 4c, the LCD touch screen, switch button, charging port, and external communication interface are installed on the upper shell. The device uses a TJC8048X570_011C touch screen (Shenzhen Taojingchi Electronics Co., Ltd) for control and display of results. The touch screen interactive display has a resolution of 1024 × 600 and 128 M flash memory. The universal serial bus (USB) interface can be used to output real‐time measurement results during the LAMP amplification process.

The POCT device employs STM32F103C8T6 as the main controller, which communicates with the touch screen via a UART (universal asynchronous receiver/transmitter) interface to facilitate user interaction and control [34]. The system communicates with the TCS34725 color sensor using the IIC (interintegrated circuit) interface for sensor configuration and the acquisition of measurement data. The temperature signal is sampled using an ADC, and the heater’s operation is modulated by a pulse‐width modulation (PWM) signal to ensure precise temperature regulation. The rotational speed of the two‐phase direct current (DC) stepper motor is governed by a PWM signal transmitted through the DM420 stepper motor driver, while the motor’s direction of rotation is controlled via input/output (I/O) signals. The interconnections and functional relationships among the subsystems and the main controller are depicted in Figure 4d.

In accordance with the technical requirements for microfluidic chip nucleic acid isothermal amplification instruments specified in GB/T 41,407–2022, the isothermal amplification temperature in POCT is preset at 65°C, and the reaction time is preset at 20 min by default. Additionally, to accommodate the varying times and temperatures required for different application, the reaction temperature and time can be adjusted as needed.

The control circuit board in the system is manufactured by JLCPCB (Shenzhen, China). The bill of materials (BOM) in the system assembly are available in Table S1. The total cost of the entire device is $108.1.

Figure S4 a shows the finished POCT device, while Figure S4 b shows the POCT device in operation.

### 2.9. Workflow of the POCT

When carrying out sample testing using the designed POCT device and microfluidic chip, first add the sample to the lysis chamber of the microfluidic chip. Then, turn on the power and click “START” on the touchscreen to begin the detection process. Figure S3 shows the operation process of the device. The device will then automatically open the door, allowing the microfluidic chip loaded with the sample to be positioned precisely in the alignment slot on the POCT heating plate.

First, the system turns off all light sources in order to measure and collect the dark‐field background noise signal of the optical sensor. It then enables the high‐uniformity white light source and positions the whiteboard on the magnetic bead transfer device within the sensor’s detection area. It then performs white balance calibration, calculates and records the dark noise compensation coefficient and white balance correction parameters, and provides a reference for subsequent color measurements. Conduct the first color measurement simultaneously.

Next, the magnetic bead transfer device moves the magnetic beads horizontally back and forth at a speed of 3 mm/s within a range of 4 mm in the lysis chamber. This involves moving left once, pausing for 1 s, and moving right once. This sequence is repeated 30 times, taking ~4 min. This process fully achieves sample lysis and efficiently adsorbs nucleic acid onto the surface of the magnetic beads to form a magnetic bead nucleic acid complex. The magnetic bead transfer device then transfers the complex from the lysis chamber to the amplification chamber at a speed of 0.5 mm/s.

**Figure 6:**
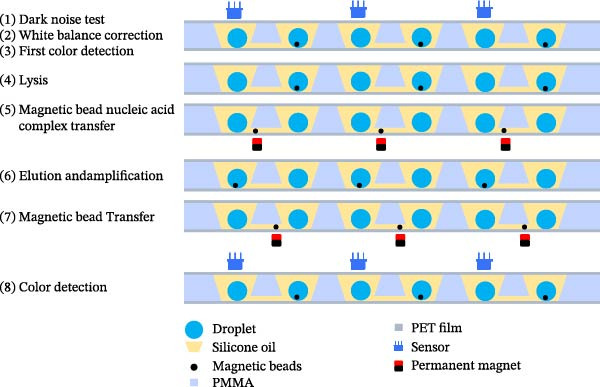
Sample lysis, nucleic acid transfer, amplification and detection process.

Once the transfer is complete, the system will start the heating program heating the reaction system to 65°C at a rate of 6°C/s for a 20 min constant temperature amplification reaction. Once the amplification has finished, the device activates the cooling fan, which cools the aluminum heater to room temperature within 2 min. At the same time, the magnetic bead transfer device removes the magnetic beads from the lysis chamber to reduce their impact on color measurement. Then, the system activates the white light source, collects color signals from the magnification chamber, and determines the final result by measuring the color difference before and after the reaction. Figure 6 shows the process of sample lysis, nucleic acid transfer, amplification, and detection.

### 2.10. Aerosol Contamination Prevention Measures

To address the risk of aerosol contamination inherently associated with LAMP, this study implemented a multi‑level contamination prevention strategy. The core innovation lies in the closed‑tube detection design: the microfluidic chip is fabricated as a sealed chamber structure, in which nucleic acid extraction, amplification, and colorimetric detection are all completed without ever opening the chip. Silicone oil is used to physically isolate different chambers within the chip, effectively preventing the migration of amplification products between chambers. The color sensor directly reads the colorimetric signal through the transparent window of the chip, eliminating the need to open the chip or transfer reaction solutions, thus completely avoiding exposure of amplicons to the environment. Automated operation further minimizes manual intervention; the device integrates magnetic bead transfer, temperature control, and color detection functions, operating fully automatically from sample loading to result output, thereby eliminating manual pipetting or opening steps that could generate aerosols. Moreover, the microfluidic chip is designed as a disposable consumable and is discarded after a single use, avoiding cross‑contamination risks associated with chip reuse. Stringent negative controls are also employed; a no‑template control is included in each batch of experiments; if the no‑template control yields a positive signal, indicating reagent or environmental contamination, the entire batch is discarded and a contamination investigation is immediately initiated. During method validation, no positive signals are observed in any no‑template control, confirming the effectiveness of the contamination prevention system.

In addition, experimental operations strictly adhere to the principle of molecular diagnostic laboratory zoning. Each zones equipped with independent ventilation systems and consumables to prevent cross‑contamination, and personnel, samples, reagents, and consumables all follow a unidirectional flow principle, with reverse flow strictly prohibited, thereby eliminating contamination pathways by design. Finally, as an additional aerosol suppression measure, after amplification the device automatically heats the reaction chamber to 95°C for 10 minutes to inactivate amplification products, further reducing contamination risks during any subsequent handling.

## 3. Results

### 3.1. Validation of Nucleic Acid Extraction and Transfer Efficiency

To verify the effects of the microfluidic chip and POCT on nucleic acid extraction and transfer, nucleic acids are extracted from samples using microfluidic chips with the POCT and conventional magnetic bead‐based instruments. The absorbance of the extracted nucleic acids is then measured at 260 and 280 nm using a UV spectrophotometer. The results are shown in Table 3.

**Table 3:** Comparison of nucleic acid extraction effects.

ASFV	Degree of automation	Nucleic acid extraction concentration (ng/μL)	A260	A280	A260/A280
Microfluidic chips	Automatic	149.16 ± 1.79	2.21 ± 0.06	1.21 ± 0.06	1.81 ± 0.01
Centrifugal tubes	Manual	166.36 ± 1.61	3.29 ± 0.11	1.76 ± 0.06	1.86 ± 0.01

The purity of the nucleic acid extraction process can be validated by measuring the absorbance ratio of the nucleic acid solution at 260 nm (A260) and 280 nm (A280). An A260/A280 ratio of around 1.8 indicates minimal protein contamination and good purity of the extracted nucleic acids, meeting the requirements for subsequent detection. Most importantly, the microfluidic chip is designed to integrate the entire nucleic acid “extraction‐transfer” process with POCT, achieving full automation and successfully eliminating the cumbersome manual steps required by traditional methods. Simultaneously, the entire process does not require complex external equipment, thereby simplifying the entire operational workflow. The high consistency of the data in Table 3 demonstrates that this integrated system does not cause significant nucleic acid loss during transfer and has high transfer efficiency.

The concentrations of nucleic acids extracted using microfluidic chips and POCT are comparable to those obtained through traditional methods. This shows that our microfluidic structure is effective at achieving cell lysis and nucleic acid capture.

### 3.2. Stability Validation of Colorimetric Assays

The system uses TCS34725 color sensors to detect LAMP results. To enhance color detection accuracy, a constant‐current source is used to drive the light source and ensure consistent light intensity. The system also automatically performs background detection and white balance calibration before each measurement. Once background detection and white balance have been completed, the system uses the absolute change in color difference between the before and after the reaction measurements to determine the reaction results. The chamber layer of the microfluidic chip is made from white PMMA to minimize light scattering and absorb background noise. Finally, the top and bottom sealing films are made from light diffusion materials to improve light uniformity and prevent uneven light distribution. Together, these methods enhance the sensitivity of colorimetric detection.

The accuracy and repeatability of the integrated color detection module are evaluated using three standard dyes: indigo carmine (1 μmol L^−1^), bromocresol green (1 μmol L^−1^), and ponceau red (1 μmol L^−1^). These are used as quantitative color standards. First, 49 μL of ultrapure H_2_O is added to each of the nine amplification chambers. Subsequently, 1 μL of ponceau red is added to amplification chambers 1, 2, and 3 of the microfluidic chip, 1 μL of bromocresol green to chambers 4, 5, and 6, and 1 μL of indigo carmine to chambers 7, 8, and 9 of the microfluidic chip. This simulates the color distribution observed in actual detection. During colorimetric measurements, follow the standard LAMP detection protocol to obtain three sets of R, G, and B color difference values per measurement. Ten independent complete detection cycles are performed, with repeat measurements conducted for each dye chamber, yielding a total of 30 valid R, G, B data sets. As shown in Table 4, the detailed RGB values and calculated CVs from 30 repeated measurements (10 independent detection cycles × 3 parallel chambers per dye) using three standard dyes. The CV values for all channels are consistently below 0.05% (R: 0.05%, G: 0.04%, B: 0.03–0.04%), demonstrating excellent repeatability and stability of the colorimetric detection module.

**Table 4:** Repeatability validation results of the colorimetric detection module using three samples (*n* = 30, including 10 independent runs).

Sample	Channel	Measurement mean ± standard deviation	CV (%)
Indigo carmine	R	218.3 ± 0.11	0.05
	G	48.6 ± 0.02	0.04
	B	37.2 ± 0.01	0.03
Bromocresol green	R	55.4 ± 0.031	0.05
	G	207.8 ± 0.08	0.04
	B	68.1 ± 0.03	0.04
Ponceau red	R	42.3 ± 0.02	0.05
	G	48.9 ± 0.02	0.04
	B	195.6 ± 0.08	0.04

Consequently, in complex microfluidic detection environments, the system exhibits minimal optical reading fluctuations and outstanding stability. This ensures the reliable interpretation of LAMP colorimetric results and fulfills a prerequisite for POCT.

### 3.3. Determination of Detection Limit

In order to accurately assess the analytical sensitivity of the designed microfluidic chip and LAMP detection device, the detection limit is determined experimentally. A recombinant plasmid containing the target ASFV‐specific gene fragment served as the standard material. A series of samples with concentrations ranging from 10^0^ to 10^4^ copies/μL are prepared using 10‐fold serial dilutions with ultrapure water. Meanwhile, establish a negative control group using ultrapure water on the microfluidic chip, and use a sample with 10^4^ copies/μL as the positive control group.

**Figure 7:**
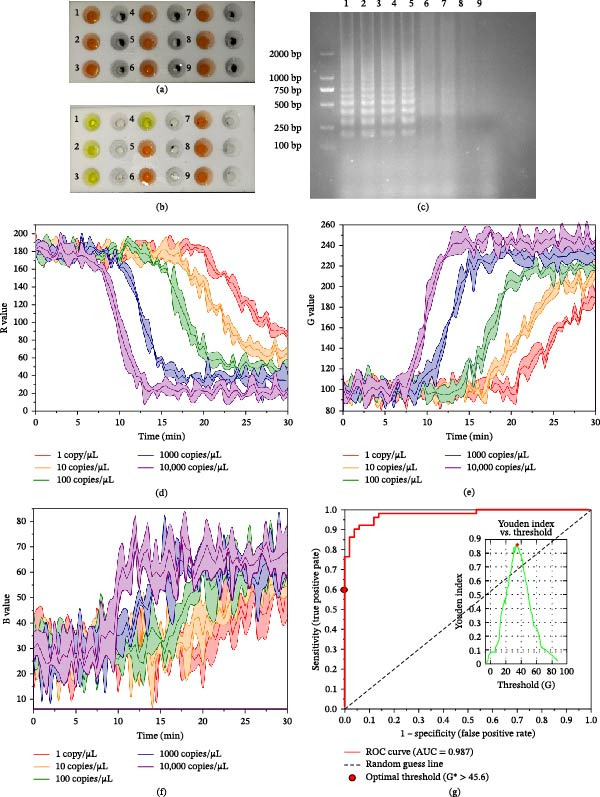
(a) Microfluidic chip before reaction, (b) microfluidic chip after reaction, (c) electrophoretic analysis results, (d) change in R value during amplification, (e) change in G value during amplification, (f) change in B value during amplification, and (g) ROC analysis.

Before determining the detection limit, first analyze the aerosol contamination blocking capability of the designed microfluidic chip. Ultrapure water is added to lysis chambers 5, 6, 7, 8, and 9 to act as negative controls. Simultaneously, add the sample with a plasmid concentration of 10^4^ copies/μL to the sample inlet of the remaining lysis chamber. Figure 7a shows status of the prereaction chip. Once the reaction program has been initiated, the system automatically carries out the entire nucleic acid extraction, transfer, and LAMP amplification process, as well as the signal detection. Visually, the positive group exhibited a yellow‐green color after the reaction, while the negative control samples showed no significant changes, as can be seen in Figure 7b.

To validate the specificity of the LAMP reaction, 10 μL pipettes are used to penetrate the PET sealing membrane and aspirate the reaction solutions from each amplification chamber, which is then analyzed by gel electrophoresis. Electrophoresis results showed specific amplification bands in all positive samples, confirming a successful LAMP reaction. The absence of amplification bands is observed in any of the negative controls, which demonstrates the absence of aerosol contamination within the reaction system, as shown in Figure 7c. Unlike plasmid amplification, diffuse bands appeared during electrophoresis due to the presence of abundant host DNA in the blood samples. PCR analysis confirmed that this is not nonspecific amplification. These results conclusively demonstrate that the silicone oil layer and the PET sealing membrane effectively prevent aerosol contamination.

To obtain reliable statistical results, eight independent replicate experiments are performed at each concentration level. During the reaction, the designed POCT is used for secondary detection, recording the changes in R, G, and B values for each chamber, as well as the R, G, and B differences before and after the reaction. Real‐time R, G, and B values from the amplification chamber are output to a computer via the instrument’s USB interface. As shown in Figure 7d–f, the R value decreased during amplification, whereas the G and B values increased. B exhibited greater variation than G, and both B and G displayed the expected amplification kinetic curve. This indicates a clear concentration‐dependent relationship between signal intensity and time to onset. Due to the consistent trend in G and B values, as well as their significant divergence from R values, the difference in B values before and after the reaction is adopted as the criterion for distinguishing between negative and positive results in subsequent studies.

The experimental results are presented in Table 5. When the concentration of the plasmid is ≥ 10^2^ copies/μL, all eight replicates yielded stable positive results, achieving a detection rate of 100%. At a concentration of 10^1^ copies/μL, detection occurred in three out of eight replicates, yielding a detection rate of 37.5%. At a concentration of 10^0^ copies/μL, all test results are negative.

**Table 5:** Distribution of testing and detection times under different concentration conditions (%).

Concentration (copies/μL)	Number of tests	Number of detections	Detection rate (%)	Negative control	Positive control
10^5^	8	8	100	Negative	Positive
10^4^	8	8	100	Negative	Positive
10^3^	8	8	100	Negative	Positive
10^2^	8	8	100	Negative	Positive
10^1^	8	3	37.5	Negative	Positive
10^0^	8	0	0	Negative	Positive

The optimal cutoff value is determined through receiver operating characteristic (ROC) curve analysis. Specifically, a ROC curve is plotted using the G difference measurements from all clinical samples plotted against their actual infection status to create an ROC curve, and the area under the curve (AUC) is calculated.

The samples used in this study are primarily laboratory‑preserved ASFV‑positive porcine blood samples, which are confirmed by qPCR and have well‑characterized viral loads. However, genuine clinical specimens present more complex matrix backgrounds and inter‑individual variations. Future studies should further validate the proposed method using a larger cohort of genuine clinical samples to more comprehensively evaluate its accuracy and reliability in real‑world applications.

The optimal cutoff point is defined as the G difference value corresponding to the maximum Youden’s index, as shown in Figure 7g. ROC analysis shows that the system has excellent discrimination performance, with an AUC of 0.987. Taking the maximization of Youden index as the standard, the optimal cutoff value was determined as G difference > 45.6. As shown in the G difference distribution histogram embedded in Figure 7g, this cutoff value forms a clear segmentation between positive samples and negative samples, which verifies its excellent discrimination efficiency. At this threshold, the system demonstrated 98.0% sensitivity and 98.1% specificity. When the plasmid concentration is no less than 10^2^ copies/μL, all replicate tests are positive, achieving a detection rate of 100%.

To objectively evaluate the performance of the developed device, its key parameters are compared with those of several typical rapid nucleic acid diagnostic platforms reported in recent studies, as summarized in Table 6.

**Table 6:** Comparative analysis of the performance of various rapid nucleic acid detection platforms.

Reference	Technology/platform	LoD (copies/μL)	Diagnostic performance	Total time (min)	Key features
This work	Microfluidic chip + LAMP + color sensor	10^2^	Sensitivity 97.6%, Specificity 98.0%	25	Fully automated, closed‐tube, 9 samples/run, low‐cost, portable, accuracy 96.6%
Zhao et al. [43]	RT‐RAA‐CRISPR/Cas12a + QDMs lateral flow	1	100% concordance with RT‐qPCR, no cross‐reactivity	<60	Multiplex, smartphone‐compatible
Mao et al. [44]	MCDA + nanoparticle LFB	200^a^	No cross‐reactivity	30	Recognizes 10 target regions, real‐time turbidity (MG) dye
Lin et al. [45]	One‐pot RPA‐Cas12a + portable device	6 (visual)	97% (ASFV)	40–60	All‐in‐one, de novo autodesigner, lyophilized reagents
Ji et al. [12]	Microfluidic‐LAMP chip	10^1^–10^2^	Specificity 100%	<60	Simultaneous detection of ASFV, PRV, PPV, PCV2, PRRSV
Dhandapani et al. [46]	Magnetic bead capture+qPCR/RPA	10^2^	Comparable to commercial kits, sequence‐specific	—	Direct capture from crude samples, SYBR Green I visual
Cao et al. [47]	Direct PCR + CRISPR/ Cas12a + LFA	4	Comparable to qPCR	—	No nucleic acid extraction, fluorescence/LFA dual mode
Xiong et al. [48]	One‐pot RPA‐Cas12a + LFA	3.07	93.3% agreement with qPCR, high specificity	40	One‐pot avoids aerosol, low cost
Wang et al. [49]	RPA + lateral flow gene assay	10^2^	No cross‐reactivity with CSFV	30	Tailed primers, AuNP‐labeled probe, no expensive instruments

*Note*: “—” indicates the parameter was not explicitly reported in the original article.

^a^Copies/reaction.

As shown in Table 6, compared with similar technologies, the developed device + achieved a lower limit of detection (10^2^ copies/μL) and a shorter assay time, while maintaining 100% sensitivity and specificity. More importantly, the device integrates sample processing, amplification, and detection into a single system, enabling truly fully automatic closed‐tube operation and avoiding aerosol contamination, which is critical for point‐of‐care testing.

### 3.4. Clinical Diagnostic Performance Evaluation

A blinded test is conducted using a cohort comprising 87 clinical samples to evaluate the clinical diagnostic performance of the designed microfluidic chip and POCT. Positive samples are obtained by diluting blood samples from infected pigs. This included 44 samples that are confirmed as positive by qPCR and 43 samples that are confirmed as negative. Thirty‐nine of these tested negative by qPCR; the remaining four tested positive for other common pathogens (CSFV, PRRSV, PEDV, and PRV) and are used to validate cross‐reactivity.

**Figure 8:**
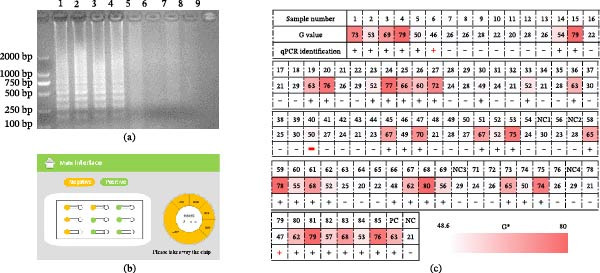
(a) Electrophoresis analysis of amplification results of clinical samples; (b) POCT output results; and (c) validation results using clinical blood samples in a blind test, showing sample G differential data obtained from SPOT device readings. “PC” indicates the positive control sample, “NC” indicates the negative control sample, “NC1” indicates CSFV, “NC2” indicates PRRSV, “NC3” indicates PEDV, and “NC4” indicates PRV.

Add positive samples to lysis chambers 1–4. Add CSFV to lysis chamber 5, PRRSV to lysis chamber 6, PEDV to lysis chamber 7, and PRV to lysis chamber 8. Add ultrapure water to lysis chamber 9 as a negative control. The microfluidic chip and POCT will then automatically perform nucleic acid extraction and transfer, followed by LAMP amplification. After the reaction, the POCT results are shown in Figure 8a. The results indicate that the ASFV‐positive sample underwent successful LAMP amplification, whereas the other samples yielded negative results. Ultrapure water, used as a negative control, also failed to amplify. Clearly, the designed microfluidic chip and nucleic acid detection device can specifically detect ASFV.

After amplification, aspirate 10 μL of reaction solution from each amplification chamber using a pipette for gel electrophoresis analysis. The electrophoresis results are shown in Figure 8b. The diffuse bands appeared during electrophoresis due to the presence of abundant host DNA in the blood samples.

The microfluidic chip designed for this purpose, along with POCT, is used to analyze the aforementioned 87 sets of known positive and negative clinical samples. The plasmid samples synthesized during testing are used as the positive control group, while ultrapure water is used as the negative control group. The results of each test are recorded, including the intensity of the detection signal and whether a positive result is indicated. These results are shown in Figure 8c. The microfluidic chip and POCT demonstrated excellent diagnostic performance. The system achieved sensitivities of 95.5% (42/44) and 97.7% (42/43), with an accuracy of 96.6% (84/87). All four of the other pathogen samples tested negative, indicating no cross‐reactivity.

Clinical test results indicate that all positive controls produced stable, high‐signal responses, as shown in Figure 8c, with signal values primarily distributed between 60 and 80. In contrast, all negative control signals remained at background levels, with signal values concentrated between 20 and 30. This clear distinction between the two groups demonstrates the high specificity and reliability of the reaction system. Furthermore, in sample tests containing other common swine pathogens, such as CSFV, PRRSV, PEDV, and PRV, the signal values detected are all below 30, showing no significant difference from the negative control group and fully demonstrating the absence of cross‐reactivity in this method. In summary, the microfluidic chip and POCT system designed in this study offer significant advantages in terms of application in complex field environments. Their outstanding target specificity avoids false positives and enables specific identification of ASFV.

## 4. Discussion

The microfluidic chip and detection system is a key innovation due to its low‐cost, high‐efficiency implementation of fully automated, simultaneous lysis, transfer, amplification, and detection of nine samples, eliminating the need for external equipment or manual operation. The system also uses colorimetric measurement to provide a portable nucleic acid analyzer with high specificity and accuracy that is unaffected by lighting conditions or smartphone camera software.

The SPOT detection method developed in this study differs from qPCR in terms of detection principle, target selection, and application scenarios. qPCR features high sensitivity and quantitative capability, making it ideal for laboratory confirmatory testing. In contrast, the proposed method, based on LAMP technology and uses a color sensor for endpoint interpretation, exhibiting superiorities in operational simplicity, assay speed (~25 min vs. 1.5–2 h), and resistance to matrix interference, making it more suitable for point‐of‐care testing scenarios. The reliability of the two methods should be evaluated in conjunction with specific application scenarios: qPCR demonstrates higher maturity under laboratory settings, whereas the developed method significantly reduces the risk of aerosol contamination in field screening owing to its high degree of automation, good repeatability (CV < 2%), and closed‐tube operation. At the same time, this method employs multiprimer recognition and a highly conserved *p10* gene target, providing better coverage of variant strains. A comparative analysis of the reliability of the two methods is shown in Table 7.

**Table 7:** Comparative reliability analysis of qPCR and the POCT.

Evaluation dimension	qPCR	This method
Sensitivity	5–10 copies/μL	10^2^ copies/μL
Specificity	High, but affected by target region mutations	High, with multiple primers reducing false‐negative risk
Repeatability	CV <5% (under standard conditions)	CV <2% (color sensor)
Inhibitor tolerance	Sensitive to inhibitors; requires purified nucleic acid	Good tolerance to blood matrix
Operational complexity	High; requires trained personnel	Low; fully automated closed‐tube operation
Time to result	1.5–2 h	25 min
Instrument cost	High (>$10,000)	Low (portable design)
Applicable scenario	Laboratory confirmatory testing	Point‐of‐care screening

To achieve high‐sensitivity color measurement, the chip features a white color chamber design to minimize light scattering and absorb background noise. The light source is driven by a high‐precision constant‐current source chip to enhance its accuracy and stability. A PET film with a light‐scattering structure is used to improve the uniformity and consistency of both incident and reflected light. In the chip, silicone oil is adopted as the continuous phase for testing due to its excellent optical transparency, which allows light to propagate freely within the microfluidic channels.

In the multivalidation of R, G, and B channels, the TCS34725 sensing module and optimized colorimetric method demonstrated excellent repeatability, with a coefficient of variation as low as 0.04%. This effectively improves accuracy and reliability, eliminating the influence of lighting conditions and imaging software on the detection results when using camera recognition.

This method enables efficient nucleic acid transfer and separation between different chambers in an economical, effective, and reliable manner. First, simple hydrophobic treatment of the microchannels and reaction chambers isolates the reaction system from the microfluidic chip. Second, the exceptional biocompatibility of silicone oil ensures no toxicity or adverse effects on biological samples such as cells and proteins. Finally, the microfluidic chip utilizes silicone oil as the continuous phase, thereby generating oil‐in‐water droplets. This enables sample lysis, transfer, detection, and isolation between different chambers, resulting in a fully integrated system from sample input to result output, eliminating the need for external equipment.

The system incorporates an effective chip architecture that seals silicone oil and film layers, preventing aerosol diffusion and nonspecific amplification. This significantly reduces false positive rates and enhances detection accuracy. The implementation of an expert PID temperature control algorithm simultaneously enhances the temperature ramp rate (6°C/s) and control precision (±0.1°C), further reducing nonspecific amplification. The result is a low‐cost, convenient, and rapid detection method with a sensitivity of 10^2^ copies/μL, which has significant clinical value in the early diagnosis of infection.

Another key innovation of the system is the development of a portable, battery‐powered, all‐in‐one rapid nucleic acid diagnostic device. It offers lower equipment and testing costs, shorter detection times, and does not require any external devices, manual operations, or specialized laboratories throughout the entire testing process. It meets national standards and achieves the ultimate balance of cost and speed without compromising accuracy. Second, the POCT device weighs just 2 kg and features a compact, lightweight, portable design. Test results can be transmitted via a USB cable to facilitate centralized data management and support test‐trace‐isolate protocols. Compared to other devices, it offers the advantages of a shorter testing time, lower cost, and the ability to process nine samples per run. Given the high frequency of testing, rapid turnaround time may be more critical than detection sensitivity.

## 5. Conclusion

The designed nucleic acid diagnostic device and microfluidic chip enable rapid diagnosis without requiring specialized technical personnel or external auxiliary equipment. Only a small liquid sample is required, which undergoes pretreatment and amplification reactions via the microfluidic chip. This low‐cost, integrated microfluidic chip can process nine samples simultaneously and enables efficient bead transfer through low‐cost hydrophobic treatment and channel parameter optimization. The measurement accuracy is improved by the design of the constant‐current light source circuit, the selection of the chamber material and color, and the use of light diffuse PET film. At the same time, an automated, portable, integrated diagnostic device is developed. With high temperature control precision (±0.1°C) and rapid heating capability (6°C/s), it can detect nucleic acids at concentrations as low as 10^2^ copies/μL when used with the designed microfluidic chip. As a rapid screening tool tailored for on‐site real‐time detection scenarios, the device complements the laboratory qPCR confirmation method in ASFV prevention and control, providing new technical support for the establishment of a hierarchical and multidimensional detection system.

The nucleic acid diagnostic system developed in this study comprises a disposable microfluidic chip and a portable battery‐powered device, enabling real‐time testing without requiring specialized training or external instruments. This truly achieves rapid point‐of‐care diagnosis with “sample in, result out.” The system’s low‐cost, lightweight, and low‐power design significantly reduces barriers to use and deployment complexity, providing an affordable solution for immediate screening of ASF. Future research will focus on optimizing calcein concentration within the system to enable semiquantitative or even quantitative analysis. Concurrently, clinical sample scale‐up and rigorous multicenter performance validation will be conducted to meet field rapid screening standards. Furthermore, the collection of real clinical samples will be expanded, and multicenter clinical validation will be carried out. The advantages and disadvantages of its application in actual clinical practice will be clarified. This will provide a scientific basis for the clinical promotion and optimization of this method. In addition, the modular design of the platform ensures excellent versatility, demonstrating broad application prospects and significant translational potential.

## Author Contributions

All content is solely developed and written by the authors.

## Funding

The authors gratefully acknowledge the funding from Chongqing Special Key Projects for Technological Innovation and Application Development (Grant cstc2021jscx‐dxwtBX0009), National Center of Technology Innovation for Pigs (Grant NCTIP‐XD/B11), and Chongqing Special Project for Technological Innovation and Application Development (Grant CSTB2025TIAD‐qykjggX0018).

## Conflicts of Interest

The authors declare no conflicts of interest.

## Supporting Information

Additional supporting information can be found online in the Supporting Information section.

## Supporting information


**Supporting Information** Additional supporting information can be found online in the Supporting Information section. Figure S1. Test results for sealing and isolation performance of hydrophobically treated microfluidic chips. Figure S2. Microscopic images of magnetic bead transfer on a microfluidic chip: (a) before and (b) after the process. Figure S3. Interface design of the POCT. (a) initial interface, (b) door opening interface, (c) waiting for chip insertion interface, (d) nucleic acid detection interface. Figure S4. (a) The completed POCT device (b) The POCT device in operation. Table S1. Components, quantities, costs, and procurement sources included in POCT.

## Data Availability

The data that support the findings of this study are available from the corresponding author upon reasonable request.
