# Supplementary material for: A Microfluidic Chip and a Portable Colorimetric Detection Device for the Rapid, Low‐Cost, and Accurate Diagnosis of ASFV
Source: Transbound Emerg Dis. 2026 Jun 18;2026:9794229. doi: 10.1155/tbed/9794229 (PMC13277774; doi:10.1155/tbed/9794229)
Supplement: Supplementary file 1 — Supporting Information Additional supporting information can be found online in the Supporting Information section. Figure S1. Test results for sealing and isolation performance of hydrophobically treated microfluidic chips. Figure S2. Microscopic images of magnetic bead transfer on a microfluidic chip: (a) before and (b) after the process. Figure S3. Interface design of the POCT. (a) initial interface, (b) door opening interface, (c) waiting for chip insertion interface, (d) nucleic acid detection interface. Figure S4. (a) The completed POCT device (b) The POCT device in operation. Table S1. Components, quantities, costs, and procurement sources included in POCT. [file TBED-2026-9794229-s001.docx]

**Supporting Information**

**A microfluidic chip and a portable colorimetric detection device for the rapid, low-cost and accurate diagnosis of ASFV**


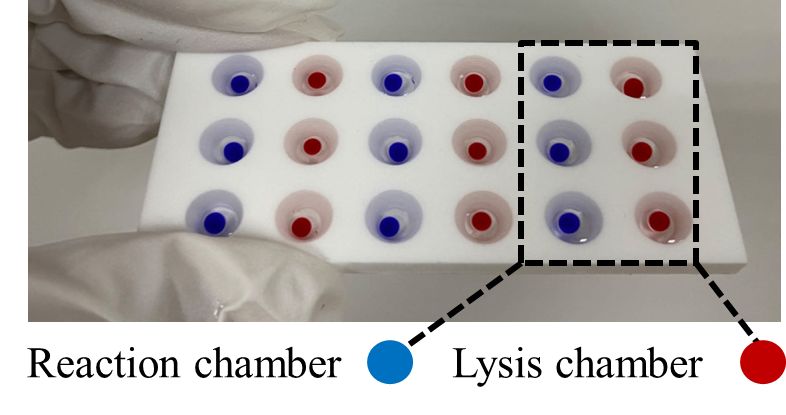


Fig. S1. Test results for sealing and isolation performance of hydrophobically treated microfluidic chips.


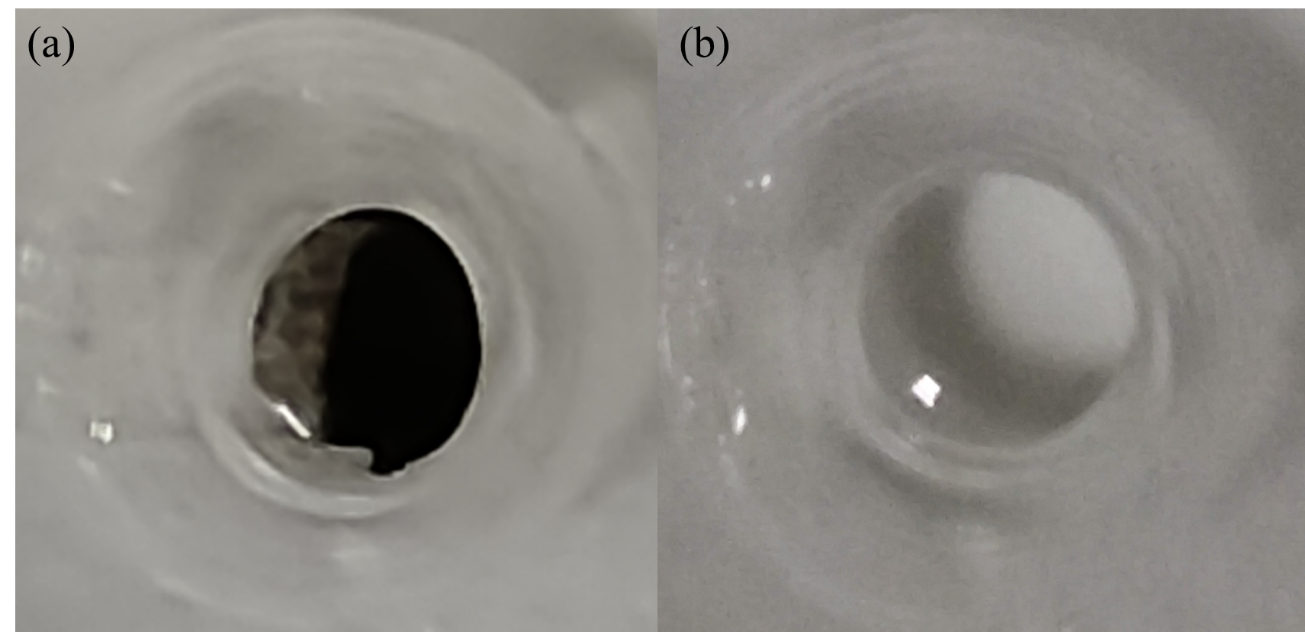


Fig. S2. Microscopic images of magnetic bead transfer on a microfluidic chip: (a) before and (b) after the process.


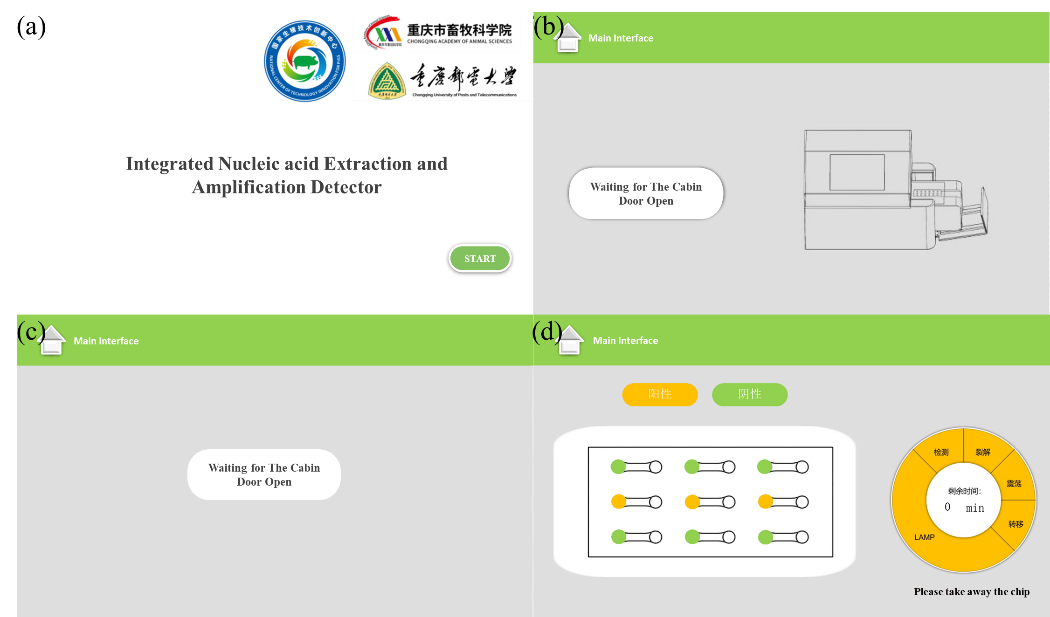


Fig. S3. Interface design of the POCT. (a) initial interface, (b) door opening interface, (c) waiting for chip insertion interface, (d) nucleic acid detection interface.


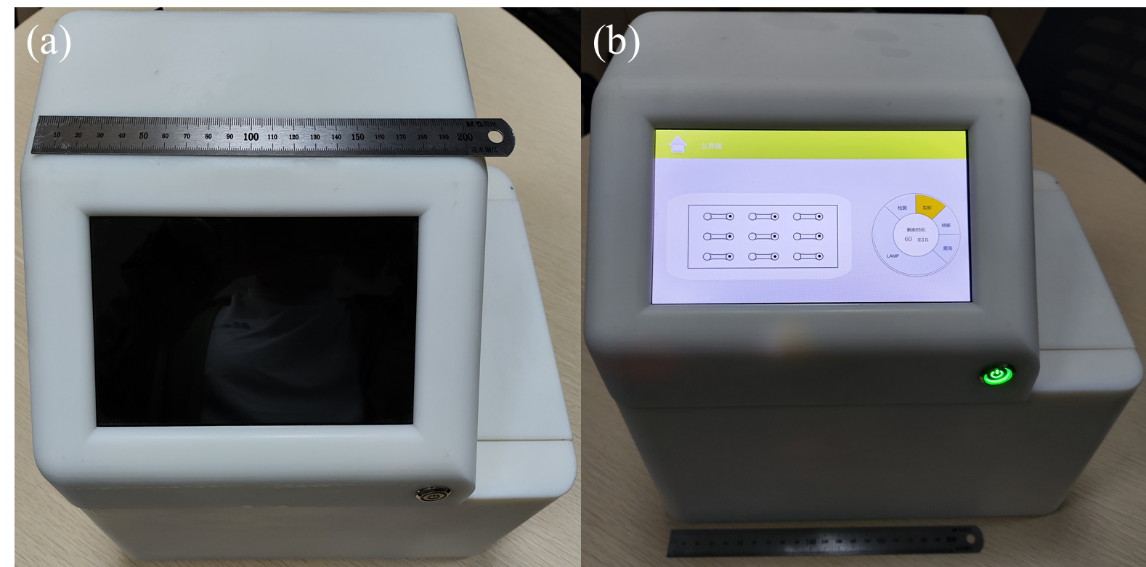


Fig. S4. (a) The completed POCT device (b) The POCT device in operation.

Table S1. Components, quantities, costs, and procurement sources included in POCT

| Item | Price($) | Quantity | Procurement sources |
| --- | --- | --- | --- |
| Stepper motor and ball screw | 15 | 2 | www.taobao.com |
| STM32 | 2 | 1 | www.taobao.com |
| Color sensor | 1 | 9 | www.taobao.com |
| Heating plate | 1.8 | 1 | www.taobao.com |
| NTC temperature sensor | 1.1 | 1 | www.taobao.com |
| Permanent magnets | 0.2 | 9 | www.taobao.com |
| Touch screen | 25 | 1 | www.taobao.com |
| Temperature controller | 10.2 | 1 | www.taobao.com |
| FR4-circuit board | 1.2 | 1 | www.taobao.com |
| Other electronic devices | 4 | 1 | www.taobao.com |
| Device housing | 10 | 1 | www.taobao.com |
| Battery | 12 | 1 | www.taobao.com |
| Total price | 108.1 | |  |
